# Correlation of left atrial function and pulmonary edema in patients with left heart failure on cardiopulmonary ultrasonography

**DOI:** 10.3389/fcvm.2023.1274443

**Published:** 2023-10-27

**Authors:** Hong Li, Ping-Xiang Hu, Jian Chen

**Affiliations:** ^1^ Department of Ultrasound, The Fourth Clinical Medical College of Guangzhou University of Chinese Medicine (Shenzhen Traditional Chinese Medicine Hospital), Shenzhen, China; ^2^ Department of Ultrasound, The First Affiliated Hospital of Shenzhen University (Shenzhen Second People’s Hospital), Shenzhen, China

**Keywords:** pulmonary ultrasound, echocardiography, heart failure, pulmonary edema, left atrial function

## Abstract

**Objective:**

Patients with heart failure with pulmonary edema may have declining left atrial (LA) function. Left atrial strain (LAS) imaging enables quantitative assessment of LA function. The aim of this prospective study was to assess the LA function and pulmonary edema in patients with heart failure evaluated by cardiopulmonary ultrasonography.

**Methods:**

Two-dimensional speckle-tracking echocardiography for LAS was performed in 115 consecutive patients with congestive heart failure. A semiquantitative B-lines score of pleural effusions was derived by pulmonary ultrasound almost at the same time by combined cardiopulmonary ultrasound.

**Results:**

Compared with those who did not have pulmonary edema, patients with pulmonary edema had lower LAS (LAS_reservoir_, 21.5 ± 4.9% vs. 9.2 ± 3.7% [*P *< 0.001]; LAS_conduit_, 10.7 ± 3.5% vs. 5.1 ± 2.1% [*P *< 0.001]; LAS_pump_, 11.3 ± 5.4% vs. 4.0 ± 2.7% [*P *< 0.001]), lower LVEF, TAPSE; and higher SPAP, E/e′, larger LA, LV, RV; more severe MR. However, there were no signiﬁcant between-group differences with respect to sex and body surface area. In patients with pulmonary edema, B-lines score was independently associated with LAS_reservoir_ (*R* = −0.71, *P *< 0.001); LAS_pump_ (*R* = −0.66, *P *< 0.001) and LAS_conduit_ (*R* = −0.56, *P *< 0.001). On multiple linear regression, decreased LAS_reservoir_ (beta = −0.61, *B* = −0.71, *P *< 0.001) and elevated SPAP (beta = 0.31, *B* = 0.13, *P *= 0.01) were significantly associated with B-lines score in heart failure.

**Conclusion:**

Declining LA function, especially the reservoir function, assessed by speckle-tracking echocardiography is related to the degree and occurrence of pulmonary edema in patients with left heart failure.

## Introduction

Pulmonary edema is one of the most serious consequences of left heart failure. It is the main determinant of adverse outcomes, higher risk of hospitalization, and emergency department visits, and is evaluated as a treatment target in patients with heart failure (1). In patients with left heart failure, when fluid filtration increases due to left ventricular (LV) filling pressure, increased extravascular lung water (EVLW) results in cardiogenic pulmonary edema. B-lines detected by lung ultrasound have been demonstrated to change dynamically with EVLW content (2). Studies have shown that pulmonary ultrasound can allow for semi-quantitative evaluation of the degree of pulmonary edema and shows a good correlation with x-ray extravascular pulmonary water score (3, 4).

The main role of the left atrium (LA) is to regulate left ventricular filling and cardiovascular function. As a complex process, the LA acts as a reservoir during systole, a conduit for direct blood flow from the pulmonary veins into the left ventricle during the early diastolic period, and as an active pump in the late diastolic period of the left ventricle. LA function has been shown to predict left ventricular filling pressure and adverse cardiac outcome (5). Decline in LA function in patients with heart failure may induce the typical symptoms of heart failure (6). Speckle tracking echocardiography (STE) is a novel imaging tool that can objectively quantify the contributions of reservoir, conduit, and active pump functions of the LA. The role of LA function as a biomarker is increasingly being evaluated by strain analysis.

However, there is a paucity of studies evaluating the prevalence and correlation of left atrial dysfunction in patients with heart failure who have pulmonary edema. In this study, we used cardiopulmonary ultrasonography for comprehensive and simultaneous assessment of the degree of pulmonary edema, left atrium strain, and other echocardiographic parameters in patients with congestive heart failure to explore the correlation of these indices with cardiogenic pulmonary edema.

## Materials and methods

### Baseline characteristics

**Figure 1:**
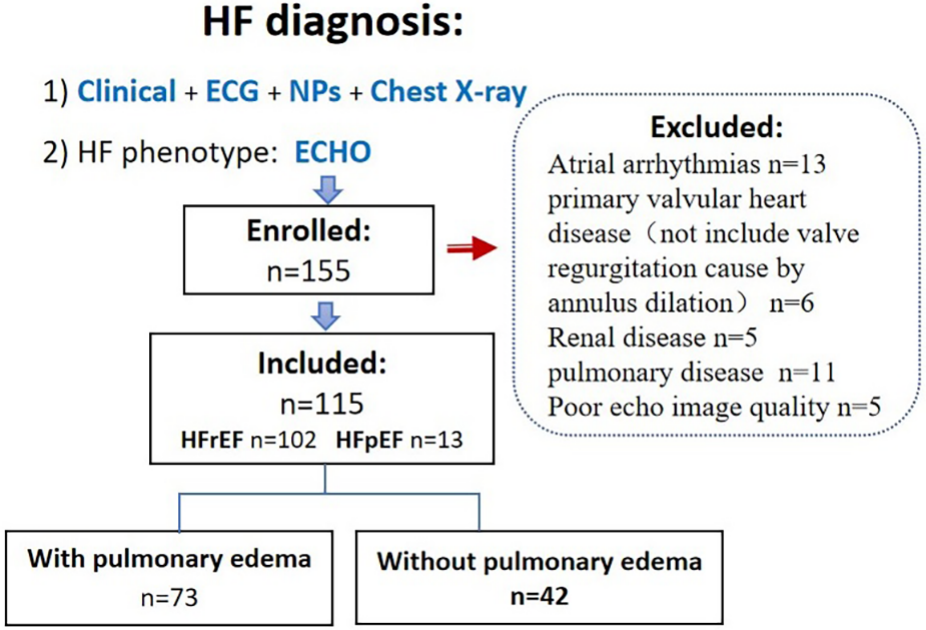
Flowchart of patient selection. HF, heart failure.

This was a prospective study conducted at an affiliated hospital of a medical college. Patients with congestive heart failure [diagnosis based on the 2016 ESC Guidelines (7)] who were admitted to our Internal Medicine-Cardiovascular Department between October 2020 and August 2022 were eligible for inclusion in this study. The Ethics Committee of the Shenzhen Traditional Chinese Medicine Hospital approved this study, and all participants provided written informed consent. The exclusion criteria were: age < 18 years, primary valvular heart disease, atrial arrhythmias, systemic disease associated with pleural edema (such as renal disease), and pulmonary disease (Figure 1).

### Cardiopulmonary ultrasonography

Examinations were performed by qualified sonographers using the same equipment (Philips APEC 7C, Philips Healthcare, Bothell, Washington, USA) and transducers (S5-1 phased-array probe [1–5 MHz] and L12-3 linear probe [3–12 MHz]). Cardiopulmonary ultrasonography was performed prior to initiation of intravenous diuretic therapy. Echocardiography was performed in the left decubitus position, supine, or near-to-supine position. Pulmonary ultrasound was performed with patients in supine or near-to-supine position.

#### Echocardiography

Two-dimensional, M-mode, and Doppler measures and indices were obtained using standard techniques outlined by the American Society of Echocardiography (8). Assessment of LV diastolic function grade included the following variables: mitral flow velocities, mitral annular e′ velocity, E/e′ ratio (Peak E wave was measured from the apical 4-chamber in Doppler velocities of early diastolic flow, and a 1.5 mm sample volume was placed at the septal and lateral corner of the mitral annulus; early diastolic velocity e′ was measured; average E/e′ ratio was then calculated), peak velocity of TR jet, and LA maximum volume index (9).

**Figure 2:**
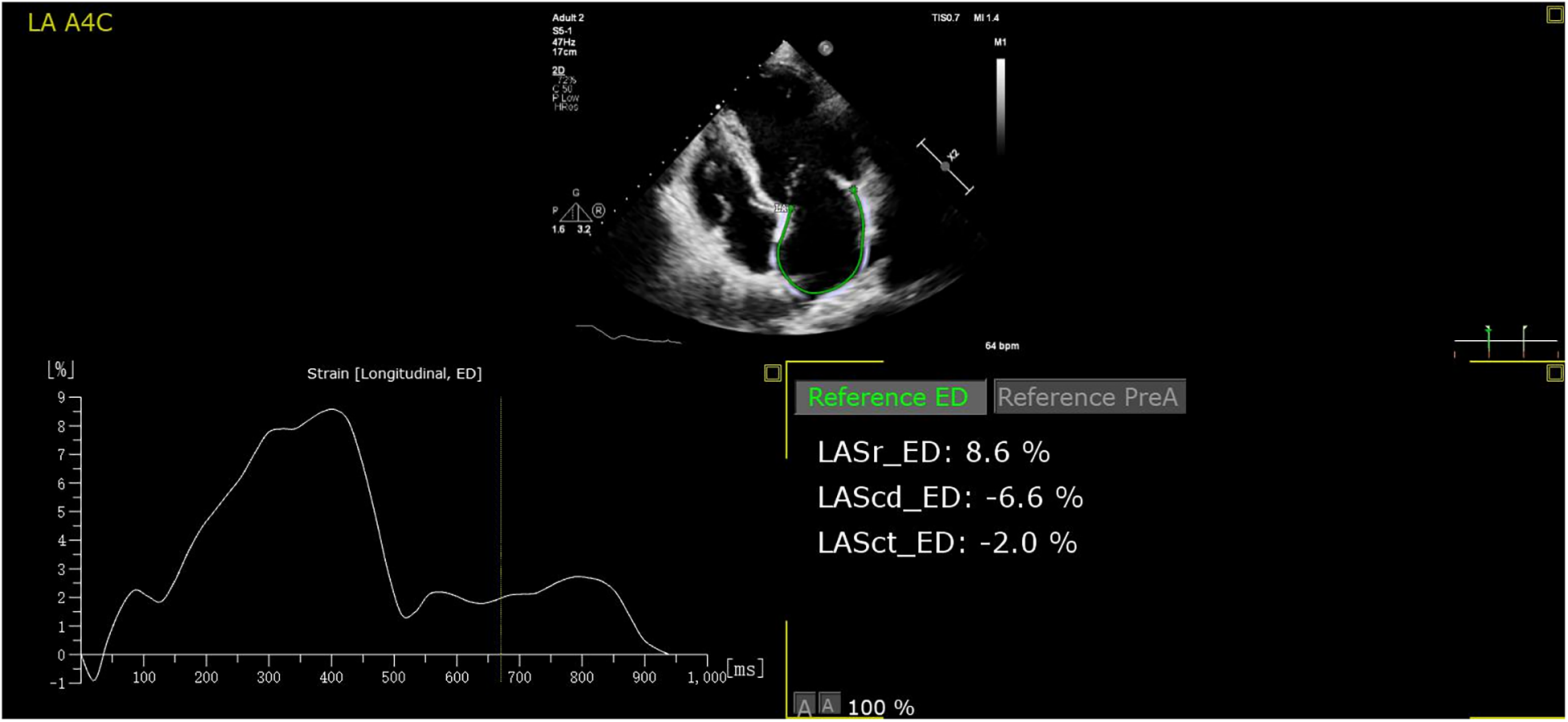
The auto strain LA algorithm acquires the LA strain parameters at both end-diastole automatically. LASr, LAS_reservoir_; LAScd, LAS_conduit_; LASct, LAS_pump_.

Three-heart-cycles were recorded to obtain images suitable for strain analysis. LAS analysis was performed on the Apical four- and two-chamber views with a frame rate ≥40 frames/s. Surface tracing was automatically generated by the system. Tracking was considered satisfactory if it covered the entire LA wall and with visible motion of the speckles; segments that failed to be tracked by the software were adjusted manually as required. Strain analysis was performed in all studies with adequate image quality. The automated strain detection measures LA conduit strain (LAS_conduit_, a “positive” wave describing LA myocardial lengthening during passive conduit function); LA pump strain (LAS_pump_, a contraction shown as a “negative” strain); and LA reservoir strain (LAS_reservoir_, “total” strain when the LA has the maximum volume) at both end-diastole (10) (all the parameters of strain refer only to the absolute value). Offline strain analysis was performed using the Auto Strain LA algorithm (QLAB version 13.0; Philips Healthcare, Andover, MA) (Figure 2). All strain measurements were performed by two investigators. Reproducibility was assessed using a random sample of 20 patients.

#### Pulmonary ultrasonography

After echocardiography, all patients underwent pulmonary ultrasonography with the S5-1 transducer for detection of B-lines. When multiple B-lines were observed, an L12-3 linear probe (3–12 MHz) was used to observe the pleural lines (smooth and regular hyperechoic linear structure in cardiogenic pulmonary edema) with a linear-probe. Abnormal pleural lines can also generate B-lines such as due to interstitial lung disease (11) or pneumonia (12, 13); thus, patients in whom abnormal pleural lines were observed by linear probe were excluded.

**Figure 3:**
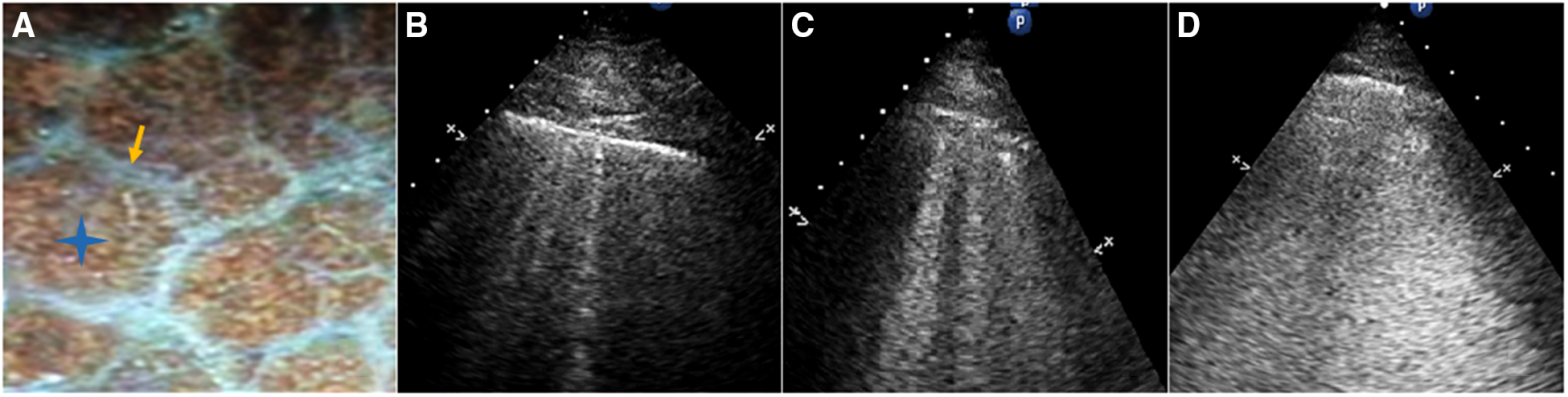
(**A**) Gross anatomy of the lung surface: the white line pointed by the yellow arrow is the subpleural interlobular septum which is approximately 7 mm apart. The blue star indicates the location of the alveoli. (**B**) Septal syndrome: B-lines are approximately 7 mm apart, corresponding to subpleural interlobular septa. (**C**) Interstitial-alveolar syndrome: B-lines are confluent. (**D**) White lung. B-lines have coalesced, resulting in an almost completely white echographic image.

The following B-line scoring method was used: The anterior and lateral chest wall was divided into seven-zones. B-line score was then calculated according to the degree of pulmonary edema (Figure 3). A score of 0 (zero) was defined as absence of B-lines. A score of 1 corresponded to septal syndrome (B-lines at regular distances, corresponding to pleural projection of the subpleural septa, at approximately 7 mm distance). Interstitial-alveolar syndrome was assigned a score of 2 (B-lines more confluent, separated by <7 mm). A score of 3 referred to white lung (B-lines coalesced, confluent B-lines >80%, resulting in an almost completely white image). Images with the highest score in each zone were recorded. A final ultrasound B-lines score was obtained by summing the B-lines scores of the seven zones of anterolateral chest scan (3).

### Statistical analysis

Normally distributed continuous variables are expressed as mean ± standard deviation, while those with skewed distribution are expressed as median (range). Categorical variables are expressed as frequency (percentage). Between-group differences were assessed using unpaired *t* test, Wilcoxon rank-sum test, Chi-squared or Fisher's exact test as appropriate. Simple linear regression was used to assess relationship of B-lines score with LA reservoir, conduit, and pump strain and other echocardiographic and clinical parameters. Stepwise multivariate logistic regression analysis was applied to identify the independent predictors of pulmonary edema. All variables associated with *P* values <0.05 were included in multivariable analysis, and collinearity between dependent factors was checked. Model performance of LAS for detecting pulmonary edema was assessed using area under the curve (AUC) along with its 95% confidence interval (CI). Intra- and inter-observer variability for LA strain were assessed using analysis of limits of agreement and the intraclass correlation coefficient in 20 patients randomly selected from the study population by the same observer (H.L.) and a second observer (J.C.). Both observers were blinded to the results of pulmonary ultrasound assessments at the time of strain analysis. Analysis was performed using SPSS 20.0 software (IBM-SPSS Inc., Chicago, IL, USA). *P* values <0.05 were considered indicative of statistical significance.

## Results

### Baseline characteristics

Of the 155 consecutive patients screened, 13 patients were excluded because of atrial arrhythmias, 11 because of concomitant lung disease, 6 because of primary severe valvular heart disease, 5 because of renal failure, and 5 because of poor echocardiographic windows. Therefore, 115 patients (mean age, 67 ± 11 years; 30.4% female) qualified the eligibility criteria. Eleven patients had HFpEF (LVEF > 50%) and 104 patients had HFrEF; among patients with pulmonary edema (*n* = 73), four patients had HFpEF. The majority of patients had coronary heart disease (52%) and hypercholesterolemia (52%), while 20% patients had type Ⅱ diabetes mellitus, and 32% patients had hypertension. Median (range) values of LA reservoir, conduit, and pump strain were 11.6 (2.9–30.8), 6.1 (1–19.4), and 5.1 (0.3–18.8), respectively. The baseline characteristics of the study population are summarized in Table 1.

**Table 1:** Characteristics of the study population.

Variable	Mean ± SD/N (%)
Age, years	63.4 ± 13.9 (28–89)
Female, *n* (%)	35 (30)
BSA, kg/m^2^	1.8 ± 0.2
NYHA class Ⅲ–Ⅳ	51 (44)
B-lines score	7 (0–20)
NT-proBNP (pg/ml)	4,043 (102–35000)
Echocardiographic data	
LVEDD, mm	59.7 ± 7.0
LVEDV_index_, ml	91 (52–171)
LA_index_, ml	34 (14–97)
Average E/e′	15.7 (5.3–42.2)
LVEF, %	38 (20–59)
RV, mm	34 (24–49)
TAPSE, mm	18 (7–27)
SPAP, mmHg	40 (27–64)
GLS, %	9.4 (3.0–28.7)
LA strain tracking echocardiography	
LAS_reservoir_, %	11.6 (2.9–30.8)
LAS_conduit_, %	6.1 (1–19.4)
LAS_pump_, %	5.1 (0.3–18.8)

BSA, body surface area; LVEF, left ventricular ejection fraction; NYHA class, New York heart association functional classification; LVEDD, left ventricular end diastolic diameter; LVEDV_index_, left ventricular end diastolic volume index; LA_index_, left atrial volume index; LVEF, left ventricular ejection fraction; RV, right ventricle; TAPSE, tricuspid annular plane systolic excursion; SPAP, systolic pulmonary artery pressure; LA, left atrial; LAS_reservoir_, left atrial strain reservoir; LAS_conduit_, left atrial strain conduit; LAS_pump_, left atrial strain pump.

### Parameters in groups with and without pulmonary edema

Patients were divided into two groups based on the presence or absence of pulmonary edema according to the pulmonary ultrasonography findings. Patients in the pulmonary edema group had bilateral septal syndrome, interstitial-alveolar syndrome, or white lung (14). Patients with pulmonary edema were significantly older and had higher NT-proBNP levels and NYHA class (Table 2).

**Table 2:** Baseline characteristics for groups with and without pulmonary edema.

	Without pulmonary edema (*n* = 42)	With pulmonary edema (*n* = 73)	*P* value
Age, years	59.5 ± 12.0	65.7 ± 14.4	0.022
Female, %	12 (28.6)	23 (31.5)	0.742
BSA, kg/m^2^	1.8 ± 0.1	1.8 ± 0.2	0.935
NT-proBNP, pg/ml	1,516 ± 1014	8,435 ± 8474	<0.001
NYHA Ⅲ–Ⅳ (*n*, %)	2 (4.8)	49 (67.1)	<0.001
Pulmonary ultrasound			
B-lines score	0	9.8 ± 3.5	
Conventional echocardiography			
LVEDD, mm	58	60.1 ± 7.6	0.002
LVEDV_index_, ml	86.2 ± 20.5	97.4 ± 25.6	0.018
LA_index_, ml	35.3	41.2	<0.001
E/e′	10.5	19.5	<0.001
LVEF, %	46	33	<0.001
RV	32	35.0 ± 4.9	0.003
TAPSE, cm	19.8 ± 5.9	17.0 ± 3.8	<0.001
PASP, mmHg	26.8 ± 7.0	43.9 ± 10.0	<0.001
GLS	12.1 ± 4.7	8.8 ± 3.2	<0.001
MR			<0.001
None	2	1	
Mild	35	28	
Moderate	2	31	
Severe	3	12	
LV diastolic function			<0.001
Grade I	27	7	
Grade II	12	26	
Grade III	3	40	
LA strain tracking echocardiography			
LAS_reservoir_, %	21.5 ± 4.9	9.2 ± 3.7	<.001
LAS_conduit_, %	10.7 ± 3.5	5.1 ± 2.1	<.001
LAS_pump_, %	11.3 ± 5.4	4.0 ± 2.7	<.001

BSA, body surface area; LVEF, left ventricular ejection fraction; NYHA class, the New York Heart Association functional classification; LVEDD, left ventricular end diastolic diameter; LVEDV_index_, left ventricular end diastolic volume index; LA_index_, left atrial volume index; LVEF, left ventricular ejection fraction; RV, right ventricle; TAPSE, tricuspid annular plane systolic excursion; SPAP, systolic pulmonary artery pressure; LA, left atrial; LAS_reservoir_, left atrial strain reservoir; LAS_conduit_, left atrial strain conduit; LAS_pump_, left atrial strain pump.

Patients with pulmonary edema had significantly reduced LAS_reservoir_, LAS_conduit_, LAS_pump_ (LASreservoir, 21.5 ± 4.9% vs. 9.2 ± 3.7% [*P *< 0.001]; LAS_conduit_, 10.7 ± 3.5% vs. 5.1 ± 2.1% [*P *< 0.001]; LAS_pump_, 11.3 ± 5.4% vs. 4.0 ± 2.7% [*P *< 0.001]). Moreover, patients with pulmonary edema had lower LVEF, TAPSE; higher SPAP, E/e′, larger LA, LV, RV; and more severe MR. However, there was no significant between-group difference with respect to sex and BSA (Table 2). On stepwise multiple logistic regression analysis, LAS_reservoir_ (OR (95% CI) = 0.53[0.35–0.80], *P *< 0.01), PASP (OR (95% CI) = 1.33[1.05–1.68], *P *< 0.05) were the independent predictors of pulmonary edema.

### Association of the degree of pulmonary edema and echocardiographic measurements

This analysis only included the group with pulmonary edema. Considering that only four patients had HFpEF in this group, we analyzed all cases including HFpEF (*n* = 4) and HFrEF (*n* = 69).

B-lines score was independently associated with LAS_reservoir_ (*R* = −0.71, *B* = −0.60, *P *< 0.001); LAS_pump_ (*R* = −0.66, *B* = −0.57, *P *< 0.001); LAS_conduit_ (*R* = −0.56, *B* = −0.95, *P *< 0.001); MR (*R* = 0.26, *B* = 1.20, *P *= 0.03); PASP (*R* = 0.54, *B* = 0.23, *P *< 0.001); E/e′ (*R* = 0.51, *B* = 0.22, *P *< 0.001); LV diastolic function (*R* = 0.41, *B* = 2.05, *P *= 0.001); LA_index_ (*R* = 0.32, *B* = 0.73, *P *= 0.011); LVGLS (*R* = −0.29, *B* = −0.36, *P *= 0.014); RV (*R* = 0.31, *B* = 0.22, *P *= 0.01); TAPSE (*R* = −0.27, *B* = −0.25, *P *= 0.04); and LVEF (*R* = −0.24, *B* = −0.10, *P *= 0.04). However, B-lines score was not associated with age (*P *= 0.38), LVEDD (*P *= 0.86), or LVEDV_index_ (*P *= 0.74).

Multiple linear regression showed that decreased LAS_reservoir_ (beta = −0.61, *B* = −0.71, *P *< 0.001) and elevated SPAP (beta = 0.31, *B* = 0.13, *P *= 0.01) were significantly associated with B-lines score in heart failure (Table 3).

**Table 3:** Univariate and multiple linear regression analysis of LAS and other echocardiographic variables for pulmonary edema.

Variable	Univariate s			Multivariate		
	Unstandardized coefficient (95% CI)	Standardized coefficients β	*P*	Unstandardized coefficient (95% CI)	Standardized coefficients β	*P*
LAS_reservoir_	−0.60 (−0.75 to −0.46)	−0.71	<0.001	−0.71 (−0.96 to −0.46)	−0.61	<0.001
LAS_pump_	−0.65 (−0.88 to −0.42)	−0.66	<0.001			
LAS_conduit_	−0.95 (−1.28 to −0.62)	−0.56	<0.001			
SPAP	0.23 (0.14 to 0.32)	0.54	<0.001	0.13 (0.04 to 0.23)	0.31	0.02
Average E/e′	0.22 (0.13 to 0.30)	0.51	<0.001			
LAV_index_	0.73 (0.02 to 0.13)	0.32	0.01			
LV_GLS_	−0.38 (−0.66 to −0.10)	−0.29	0.01			
RV	0.21 (0.06 to 0.37)	0.31	0.01			
TAPSE	−0.25 (−0.48 to −0.02)	−0.27	0.04			
MR	1.20 (0.18 to 2.24)	0.26	0.03			
LVEF	−0.10 (−0.19 to −0.01)	−0.24	0.04			

CI, confidence interval. Multivariate-adjusted *R*^2^ = 0.66, *P *< 0.001. The VIF = 1.31; Tolerance = 0.76.

LAS_reservoir_, left atrial strain reservoir; LAS_conduit_, left atrial strain conduit; LAS_pump_, left atrial strain pump; SPAP, systolic pulmonary artery pressure; LAV_index_, left atrial volume index; LV_GLS_, left ventricular global *long-axis strain*; RV, right ventricle; TAPSE, tricuspid annular plane systolic excursion; MR, mitral regurgitation; LVEF, left ventricular ejection fraction.

### Performance of LAS parameters for identification of pulmonary edema

We further investigated the value of these echocardiographic indices for identifying pulmonary edema by performing receiver operating characteristics (ROC) curve analyses. Area under the curve (AUC), optimal cutoff values, and corresponding sensitivity and specificity for pulmonary edema. Among all clinical and echocardiographic parameters analyzed, LAS_reservoir_ showed the highest diagnostic accuracy. Use of LAS_reservoir_ cutoff value of less than 16.2% for pulmonary edema was associated with 97.3% sensitivity and 85.7% specificity (AUC = 0.96, *P *< 0.001). LAS_conduit_ cut-off value of less than 7.5% for pulmonary edema had 87.7% sensitivity and 85.7% specificity (AUC = 0.92, *P *< 0.001). LAS_pump_ cut-off value of less than 6.5% for pulmonary edema had 87.7% sensitivity and 81.0% specificity (AUC = 0.86, *P *< 0.001).

### Reproducibility

For assessment of inter-observer variability, two investigators (H.L. and J.C) independently performed strain measurements in a random sample of 20 patients in a blinded manner. The intraclass correlation coefficients of LAS_reservoir_, LAS_conduit_, LAS_pump_ were 0.95 (95% CI, 0.87–98), 0.91 (95% CI, 0.73–0.97), and 0.90 (95% CI, 0.74–0.96), respectively, for intraobserver variability assessment and 0.93 (95% CI, 0.84–0.97), 0.84 (95% CI, 0.64–0.93), and 0.88 (95% CI, 0.73–0.95) for interobserver variability assessment.

## Discussion

In this study, we evaluated LA strain using a strain software that was developed for the left atrium, which is different from that used only for the evaluation of left ventricle. We found that patients with pulmonary edema had reduced LAS_reservoir_, LAS_pump_, LAS_conduit_ values compared to patients with heart failure who did not have pulmonary edema. Compared to those without pulmonary edema, patients with pulmonary edema were older, had lower LVEF, TAPSE; and higher SPAP, E/e′, larger LA, LV, RV; more severe MR. On stepwise logistic regression analysis, LAS_reservoir_ and SPAP were identified as independent predictors of pulmonary edema. We further analyzed the correlation of left atrial function and other echocardiographic and clinical parameters with the degree of pulmonary edema in the group with pulmonary edema. We found a consistent reduction in LA strain levels in patients with more severe pulmonary edema; in addition, there was no association between the degree of pulmonary edema and LV dimension or volume. On multiple linear regression, decreased LAS_reservoir_ and elevated SPAP were significantly associated with the degree of pulmonary edema.

The 2016 guidelines for assessment of left ventricular diastolic function mention E/e′ as an important and easily-obtained evaluation parameter; however, it is a less accurate parameter for estimating the LV filling pressure compared to invasive catheters (15–17). Clinical conditions, such as LV hypertrophy and dilatation, severe reduction of LV systolic function, and mitral annular calcification are liable to affect the accuracy of the filling pressure estimated from E/e′ (15, 18). Left atrial volume index (LAVI) is another important indicator of diastolic dysfunction which can be acquired with high feasibility and reproducibility. It reflects the chronic elevation of LV filling pressure; however, LAVI alone cannot be used as a marker of improved diastolic dysfunction due to the slow and incomplete remodeling of LA (19). Unlike E/e′ and LAVI, analysis of LA strain parameters allows discrimination between active and passive myocardial tissue movement, independent of tethering effects and is less load dependent compared to conventional parameters for evaluation of LA function, enabling the evaluation of LA reservoir, conduit, and pump function (20). Cameli et al. measured LA reservoir and E/e′ and invasive pulmonary artery wedge pressure (PAWP) simultaneously, and found that LAS_reservoir_ rather than E/e′ was closely related to PAWP (21). LA strain is a sensitive marker of early diastolic dysfunction and can reflect LA pressure and LV filling pressure changes instantaneously (22, 23). It was recommended to incorporate it into the 2016 EACVI/ASE criteria to improve the diagnostic efficiency of diastolic dysfunction (23).

It was reported that pulmonary edema develops acutely with LA hypertension (24). When left heart fails, increased LV filling pressure causes a backward failure process. Elevated LV pressure transmits back to LA and further to the pulmonary vessels, which leads to an increased hydrostatic force, resulting in an imbalance in the Starling equilibrium. Consequently, excessive EVLW accumulates in the lung interstitium and even in the alveoli, leading to pulmonary edema and pulmonary hypertension. Changes in EVLW can be detected by lung ultrasound and semi-quantified (25). Elevated PASP leads to RV afterload, promoting progression of RV failure. This may explain the higher PASP, larger RV, decreased TAPSE, and more severe dyspnea in patients with pulmonary edema.

LA reservoir and conduit function changes with LV compliance and impaired relaxation. In the univariate analysis, the association of LA reservoir, conduit strain, and B-line score reflects the mechanism of association of pulmonary edema with increased LA pressure and tension and impaired LA compliance. Cardiogenic pulmonary edema is the decompensated stage of left heart failure. Occurrence of pulmonary edema indicates severe increase in LA pressure, which impairs the atrial reservoir and conduit functions. In addition, our study showed a better correlation between LA reservoir strain decrease and increase in B-lines score in patients with pulmonary edema. This may be explained by the greater sensitivity of LAS_reservoir_ compared to other LA function and standard diastolic markers of elevated LA pressure and left ventricular filling pressure (26). Patients with worse left atrial function, especially LAS_reservoir_, may remain at high risk of developing pulmonary edema decompensation, hospitalization, and even emergency department visits. The decrease in left atrial function should alert the clinician that such patients require diuresis and afterload reduction. In addition, patients with heart failure showing significant reduction in left atrial strain should be evaluated by pulmonary ultrasonography or chest x-ray to rule out pulmonary edema.

In our study, we attempted to highlight the decrease in the LA pump function in patients with left heart failure with pulmonary edema. Ramkumar et al. found that LA pump function is independent of LV function (27). Different from conduit function, the LA pump is not only influenced by LV end-diastolic pressure (afterload), as the left atrium must pump blood across a higher pressure-gradient in patients with heart failure, but also influenced by atrial returned blood volume (preload) as atrial contraction adds an additional 38% filling volume during the last third of diastole in the impaired relaxation group (28). Pulmonary venous return is the predominant determinant of preload. In patients with left heart failure, occurrence of pulmonary edema further reduces the blood flow from the pulmonary circulation to the LA, further aggravating the decrease in pump function.

### Limitations

First, this was a single-center study. The study population comprised exclusively of inpatients in the Department of Cardiology who could receive cardiopulmonary ultrasound evaluation. Second, sufficient image quality for strain analysis was needed, so 5 patients were excluded from our analysis. Third, we used noninvasive B-lines scores to evaluate the degree of pulmonary edema rather than invasive hemodynamic validation. Future studies should also employ invasive hemodynamic evaluation to better characterize the physiology of LA function in patients with pulmonary edema.

## Conclusion

In patients with HF, those with pulmonary edema have worse left and right heart function. The degree of pulmonary edema increases with the decrease in left atrial function. We demonstrated that LA reservoir function is more closely related with pulmonary edema compared to the other echocardiographic parameters. Therefore, non-invasively obtained LA deformation parameters may be used to predict the occurrence of pulmonary edema.

## Data Availability

The raw data supporting the conclusions of this article will be made available by the authors, without undue reservation.
